# Inequalities in the demand and unmet need for contraception among women in four regions of Ethiopia

**DOI:** 10.1371/journal.pone.0308476

**Published:** 2024-09-10

**Authors:** Tigist Shumet Wasiyhun, Nigatu Regassa Geda

**Affiliations:** 1 Center for Population Studies, College of Development Studies, Addis Ababa University, Addis Ababa, Ethiopia; 2 Health System and Reproductive Health Research Directorate, Ethiopian Public Health Institute, Addis Ababa, Ethiopia; 3 College Pharmacy and Nutrition, University of Saskatchewan, Saskatoon, Canada; Harvard University T H Chan School of Public Health, ETHIOPIA

## Abstract

**Background:**

Unmet need for family planning is a major cause of unwanted pregnancies, which may contribute to the death of mothers and children. The aim of this study is to examine inequalities in the demand and unmet need for contraception among women in four regions (i.e., Afar, Benishangul-Gumzu, Gambela and Somali regions) of Ethiopia.

**Methods:**

The study utilized data from the 2016 Ethiopian Demographic Health Survey (EDHS), collected from 3,343 women of reproductive age 15–49 years situated in these study regions. Multilevel binary and multivariable logistic regression analysis, concentration index, and multivariate decomposition analysis were employed.

**Results:**

The study revealed that women’s employment status, education level, household wealth index, total number of children ever born, and husband’s working status had a statistically significant association with the demand for contraception. Furthermore, women’s educational level, household size, wealth index and husband’s working status had statistically significant association with unmet need for contraception. The results of the concentration index indicated that illiteracy among respondents (56%), being in the richest economic status/ wealth index (41%) and non-working status of respondents (21%) contributed substantially to the inequality in the demand for contraception use. Illiteracy of the husband (197%) and the household size less than or equal to five (184%) contributed positively, but illiteracy of respondent (-249%) and unemployment status of respondents (-119%) contributed negatively to the existing inequality in unmet need for contraception.

**Conclusion:**

The findings of this study highlight the presence of unacceptably high inequality in the demand and unmet need for contraception among women in the four study regions. Policymakers should give due attention to reducing existing socio-economic inequality to address the high unmet need for family planning and increase demand for contraception in these regions. The study strongly recommends implementing multidimensional and multisectoral approaches, which will significantly reduce inequalities in the outcome variables.

## 1. Introduction

Contraception offers several benefits to women and the communities in which they live. It enables women to space out childbirths, prevent unwanted pregnancies, and reduce the risk of morbidity and mortality associated with childbirth. Additionally, it decreases the incidence of abortions. Family planning (FP) is one of the most efficient programs a government can implement [1, 2]. Estimates suggest that by meeting the unmet need for contraception and removing barriers to its use, the world could avoid 74 million unintended pregnancies, 25 million unsafe abortion, and 47 thousand maternal death that occur annually in low-and middle-income countries [3].

Unmet need for contraception refers to a situation where fecund and sexually active women do not use any form of contraceptives, even though they do not wish to have more children or want to delay their next birth [4, 5]. Unmet need for family planning contributes to high fertility rates, leading to rapid population growth [6]. It is also a major cause of unintended pregnancies and a significant factor in maternal and infant mortality [7]. Furthermore, unmet need for contraception, adversely affects women’s reproductive health. It can impact sustainable economic growth and development, and may exacerbate gender inequalities [8, 9].

Women’s limited access to contraception services remains a critical factor to the social and public health issues worldwide. In 2019, 75.7% of women of reproductive age who needed contraception services (i.e., 842 million out of 1.11 billion) used a modern contraception method. However, 270 million still had unmet need [10]. In developing countries, including Ethiopia, the number of women with an unmet need for contraception services rose to 232 million, despite their desire to use a modern contraception method [11]. The 2016 Ethiopia Demographic Health Survey revealed that the unmet need for modern contraceptive methods among married women and sexually active unmarried women in Ethiopia was 15%, and the total demand for family planning was 40%. However, there was significant variation among regions; for example, the unmet need in four study regions was 13% in Afar, 9% in Somali, 17% in Benishangul-Gumuze, and 17% in Gambella. The demand for contraceptive use was 23% in Afar, 10% in Somali, 38% in Benishangul-Gumuz and 43% in Gambella [12].

Given this, the Ethiopian government has allocated substantial resources to the implementation of reproductive health programs. These programs aim to increase the availability and quality of contraception services, reduce the unmet need for contraception from 22% to 10%, and enhance the contraceptive use among married women to 55% by 2030 [13]. Addressing the unmet need for contraception is vital not only for improving health outcomes, such as reducing maternal and infant mortality, but also for enhancing the educational and economic opportunities for girls and women [14].

The studies we found on unmet need for contraception in the four regions of Ethiopia (i.e., Afar, Benishangul-Gumuze, Gambella and Somali regions) were very limited. Moreover, the few existing studies on demand and unmet need for contraception in other parts of Ethiopia either used small sample size [15–18] or overlooked aspects of inequality. The four regions selected for this study are among the most disadvantaged and marginalized regions in Ethiopia. They suffer low socioeconomic development, poor infrastructure, and limited access to health services, including family planning (FP). However, these regions also share relatively homogeneous cultural and religious characteristics, marked by a more nomadic lifestyle [19–22]. Therefore, we chose those four regions for our study because they have low FP utilization rates and represent the most vulnerable and underserved populations in Ethiopia, which urgently need support to enhance FP utilization.

Therefore, this study aimed to examine the inequalities in demand and unmet need for contraception among women in the four regions of Ethiopia by using the decomposition of the concentration index within the context of two theoretical models: the Anderson health-seeking behavioral model and Solar and Irwin’s Commission on Social Determinants of health framework (CSDH). The study conducted a literature search to compare the models based on their scope, components, and applicability. The Anderson model identified three sets of characteristics that affect health-seeking behavior: predisposing, enabling, and need [23]. The CSDH framework distinguished between structural and intermediary determinants of health and health inequities [24]. The study analyzed the following variables: age, education, and occupation as predisposing factors; wealth index and residence as enabling factors. However, there are other socioeconomic and political contexts which are not considered in this study as variables from the CSDH framework and the Anderson model.

Furthermore, this study sought to answer the following research questions: “What are the main factors that have significantly associated with the demand and unmet need for contraception in the four regions?” and “Which of those key factors contribute most to the inequality among women in the study regions?” Given limited availability of large-scale studies in Ethiopia, this study will provide useful information for family planning (FP) program managers and policymakers at both national and regional levels. They can use the finding of this study as an input for geographic targeting, planning, monitoring and evaluation of FP programs. The findings will also be helpful in the development mitigation strategies to increase the demand for contraception and reduce the unmet need for contraception in the study regions and the country as a whole.

## 2. Methods

### 2.1. Study area

This study was conducted in four regions of Ethiopia, namely Afar, Somali, Benishangul-Gumuz, and Gambela. These four study regions share common features, which includes: they are all geographically peripheral, economically disadvantaged in terms of access to basic services, have relatively homogeneous populations with distinct cultures [25–28]. The prevalence of most Maternal and Child Health (MCH) service indicators in these study regions is much lower compared to other regions of the country. They also have higher unmet needs and low demand for contraception [12]. According to the 2020 projected population size by Central statistics Agency (CSA) of Ethiopia in 2007, the total population were 1.95 million in Afar, 6.20 million in the Somali region, 1.16 million in Benishangul-Gumuz, and 0.478 million in Gambella [29]. The proportions of the urban population were 21% in Afar, 15% in Somali, 23.6% in Benishangul-Gumuz and 36.6% in Gambella [29]. Women in formal employment accounted for 22.7% in Afar, 18.3% in Somali, 49.7% in Benishangul-Gumuz, and 41.6% in Gambella. The educational attainment of the female household population was 31.3% in Afar, 24.7% in Somali, 53.3% in Benishangul-Gumuz and 73.3% in [29].

### 2.2. Source of data and population of the study

Data from the Ethiopian Demographic Health Survey (EDHS) 2016 were used in this study [12]. A cross-sectional survey design was used, and data were collected from all regions and city administration of Ethiopia during the study period from March 2016 to July 2016. The present analysis extracted data for 3,343 women from EDHS-2016 dataset collected from the four regions of the study. The sample included married or in-union women who were sexually active and intercourse within 30 days before the data collection date. The eligibility criteria for selecting respondents were “all women between the age 15 to 49 years who was stayed overnight in the selected households either as permanent residents or as visitor on the night before the survey” [12]. The EDHS used five different questionnaires, and this analysis used data collected by woman’s questionnaires from 4680 women aged 15 to 49 years living in the four study regions. The EDHS employed a two-stage sampling method to include the eligible respondents: first, it randomly selected groups (called primary sampling units or PSUs) from the population; second, it randomly selected individuals (called secondary sampling units or SSUs) from each group. This method ensures a representative sample while lowering costs and reducing data collection difficulties [12].

### 2.3. Variables of the study

The analysis used two dependents variables: Unmet need for contraception and demand for contraception.

Unmet need for contraception is defined as the condition of sexually active and fecund women who do not use any birth control method and either do-not want any more children or wish to delay their next child. The variable was code as “1” for women with an unmet need for contraception and “0” for women who did not have unmet need for contraception.

Demand for contraception was determined by considering all women of reproductive age (15 to 49 years) who have either a met or unmet need for contraception, including sexually active unmarried women who had intercourse within 30 days before the data collection date. The variable was coded as “0” for women without a demand for contraception use, and “1” for those with demand for contraception use.

#### 2.3.1. The exposure variables are summarized in the Table 1

**Table 1:** Description of exposure variables of the study.

Name of variables	Variable Description	Coding
Husband/partner education level /women educational level	Women aged 15–49 / Husband/partner education by highest level of schooling attended or completed.	1.No education
		2.Primary educ
		3. Secondary & above
Women age	Age in completed years	1. 15–19
		2. 20–34
		3. 35–49.
Residence	Place of residence is the type of place where the respondent resides.	1.Urban
		2. Rural
Wealth Index	Composite measure of a household’s cumulative living standard	1.Poorest
		2. Poorer
		3.Midle
		4.Richer
		5.Richest
Husband/partner Age	Completed years of the length of an existence	1.15–24
		2.25–34
		3. 34–45
		4.>45
Total number of children ever born	Number of all children, both surviving and dead to all women	1.< = 3, 2. >3
Household size	The number of persons in a private household.	1.< = 3, 2.>3
Work status	Women/husband employed in the 12 months before the survey	1.Not working
		2. working

Please note that we excluded ‘region’ from the list of exposure/explanatory variable because the 2016 Ethiopia Demographic Health Survey report had already analyzed and revealed significant variation in unmet need and demand for contraception among the study regions.

### 2.4. Statistical analyses

For data cleaning and analysis, the study utilized SPSS-v26 and STATA-v17 statistical software. The analysis began with descriptive analysis to portray the respondent’s profile/ characteristics. Subsequently, a bivariate mixed-effect logistic regression analysis was conducted, including variables with a significant p-value < 0.2 in the multivariable mixed-effect logistic regression analysis to examine the association between the outcome variable and independent variables of the study. For the multivariable analysis, a p-value <0.05 with a 95% confidence interval was used to declare statistically significant associations. The best-fitting model for multilevel analysis was selected using the Intraclass Correlation Coefficient (ICC), Median Odd Ratio (MOR), Likelihood Ratio test (LR), and Criteria information test (AIC and BIC). The model with the lowest AIC value was considered better. Additionally, the study applied a decomposition concentration index to analyze the socioeconomic inequalities.

#### 2.4.1 Concentration index (CI)

Socioeconomic inequalities in specific health sector variables do exist and they can be more pronounced at certain times or differ from one country/location to another. To identify these inequalities, scholars use concentration curves; however, these curves do not provide a measure of disparity levels that can be easily compared across different time periods, nations, or localities. Therefore, the concentration curve is directly associated with the concentration index, which quantifies the degree of inequality in a health variable related to socioeconomic factors [30]. The concentration indices(CIs) are derived from their related concentration curves and are valued as twice the area between concentration curve and the 45-degree line, representing equality. If the concentration index value is zero; it indicates no socioeconomic-related inequality. When the concentration curve lies above the line of equality C(P), the concentration index conventionally takes a negative value. For example; if healthcare utilization is concentrated among the poor, the index values range between −1 and 0. Conversely, if the curve lies below the line of equality C(P^*^), a positive value is taken; for instance, healthcare utilization concentrated among the rich would result in index values between 0 and 1 [31].

Therefore, in general, the index ranges from -1 to + 1, where the sign indicates the direction of the existing association, while the magnitude indicates the strength of the relationship [32]. The concentration index (CI) can be computed by using “convenient covariance” with the formula below:

$$\text{C}=\frac{2}{\mu }cov\;\left(y_{\text{i}},R_{\text{i}}\right)$$ (I)

Where, ‘C’ represents the concentration index ‘y_i_’ denotes the health variable for which inequality is being measured, ‘μ’ is its mean, ‘R_i_’ is the i^th^ individual’s fractional rank in the socioeconomic distribution, and ‘cov (.,.)’ stands for covariance. For the weighted data, it is necessary to compute the weighted covariance and generate a weighted fractional rank [33].

Wagstaff et al. (2001) demonstrated how the health concentration index can be decomposed into distinct factors of income-related health inequality. Each contribution is a product of the sensitivity of heath relative to that factor and the degree of income-related inequality associated with that factor. This decomposition apples to every linear regression model of health (y), such as:

y=α+∑kβkxk+ε, (II)

the concentration index for *y*, C, can be written as follows:

$$C=\sum_{k}\;\left(\beta_{k}{\bar{x}}_{k}/\mu \right)\;C_{k}+GC_{\varepsilon }/\mu ,$$ (III)

In the above formula, ‘μ’ represents the mean value of ‘*y’*, ‘$\bar{x}$_*k*_’ is the mean value of ‘*x*_*k*_’, ‘C_k_’ is the concentration index for ‘*x*_*k*_’ (defined analogously to C), and ‘*GC*_*ε*_’ is the generalized concentration index for the error term (ε). Formula number III shows; ‘C’ is equal to a weighted sum of the concentration indexes of the ‘k’ regressors where the weight for ‘*x*_*k*_’ is the elasticity of ‘y’ relative to ‘*x*_*k*_’. The remaining component, represented by the last term, indicates income-related inequality in health that is not explained by systematic variation in the regressors by income, this should approach zero in a well- specified model [34].

### 2.5. Ethics approval and consent to participate

Consent to participate was obtained from the research participants during the original data collection process, adhering to the necessary ethical approval process of 2016 EDHS dataset were used. (https://dhsprogram.com/methodology/Protecting-the-Privacy-of-DHS-Survey-Respondents.cfm). For this manuscript secondary data of EDHS 2016 publicly available data were used and we sought to have administrative permission to download and use the raw data of 2016 EDHS and the permission was obtained from the ICF international/DHS program through their platform https://dhsprogram.com/data/new-user-registration.cfm.

## 3. Results

### 3.1. Respondents’ general information

Table 2 summarizes the characteristics of the study respondents. Among all respondents, 39.9% were in the age group of 25 to 34 years. A majority,67.5%, were illiterate, and most (81.5%) lived in rural area. Over half (56.1%) resided in the poorest households, and 69.4% were not employed during the survey period.

**Table 2:** Background characteristics of the respondents, N = 3343.

Characteristics	Number	Percent
**Women’s age**		
15–24	993	29.7
25–34	1334	39.9
35–49	1016	30.4
**Women Education**		
Not able to read and write	2257	67.5
Primary	738	22.1
Secondary and above	348	10.4
**Region**		
Afar	868	26.0
Somali	975	29.2
Benishangul	797	23.8
Gambela	703	21.0
**Residence**		
Urban	618	18.5
Rural	2725	81.5
**Household wealth index**		
Poorest	1875	56.1
Poorer	363	10.9
Middle	263	7.9
Richer	290	8.7
Richest	552	16.5
**Women work status**		
No	2321	69.4
Yes	1022	30.6

### 3.2. Result from bivariate mixed effect logistic regression

The result in Table 3 present the bivariate mixed-effect logistic regression model outcomes i.e., the model used to determine the bivariate relationship of each potential predictor variable with the outcomes of interest. Factor such as Women’s age, their husband’s age, women’s employment status, education status of women and their husbands, household wealth index, total number of children ever born, and place of residence were significant factors for the demand for contraception, as all those variables had a p-value < 0.2. Similarly, the results in Table 3 also indicates that women’s age, their education status, household size, their husbands’ education and employment status, households wealth index, their husbands’ ages and place of residence are important predictors of unmet need for contraception. Therefore, the study considered these eight variables to determine associations with both demand for and unmet need for contraception in the multivariable mixed-effect logistic regression.

**Table 3:** Results of bivariate mixed effect logistic regression for demand and unmet need for contraception use, Ethiopia, N = 3343.

	Demand for Contraceptive				Unmet need contraceptive			
	UOR	95%CI		P-value	UOR	95%CI		P-value
Women age								
15–24								
25–34	0.79	0.64	0.96	**0.02**	0.86	0.69	1.07	0.19
35–49	0.51	0.41	0.64	**0.00**	0.79	0.62	1.00	0.06
Women employment								
Not working								
Working	1.41	1.16	1.71	**0.00**	1.01	0.82	1.24	0.95
Women education status								
Not educated								
Primary	2.23	1.81	2.74	**0.00**	1.52	1.22	1.89	**0.00**
Secondary and above	2.1	1.55	2.83	**0.00**	0.93	0.67	1.31	0.69
Household size								
< = 5								
>5	0.93	0.78	1.11	0.44	1.29	1.07	1.56	**0.01**
Husband education								
not educated								
primary	1.32	1.06	1.64	**0.01**	0.79	0.62	1.02	**0.07**
secondary and above	1.76	1.38	2.24	**0.00**	1.1	0.85	1.41	0.47
Wealth index								
poorest								
Poorer	1.58	1.18	2.11	**0.00**	1.09	0.81	1.47	0.58
Middle	1.59	1.14	2.22	**0.01**	0.69	0.47	1.01	**0.06**
Richer	1.97	1.41	2.76	**0.00**	0.71	0.49	1.04	**0.08**
Richest	3.18	2.3	4.39	**0.00**	0.58	0.42	0.81	**0.00**
Total children ever born								
< = 3								
>3	0.68	0.57	0.8	**0.00**	1.03	0.75	0.85	0.75
Age of husband								
15–24								
25–34	0.51	0.35	0.73	**0.00**	0.56	0.38	0.81	0.00
35–45	0.5	0.35	0.72	**0.00**	0.59	0.4	0.85	0.01
>45	0.27	0.18	0.39	**0.00**	0.44	0.29	0.66	0.00
Husband working status								
Not working								
Working	1.08	0.84	1.37	0.06	0.65	0.52	0.83	**0.00**
Type of place of residence								
Urban								
Rural	0.4	0.26	0.62	**0.00**	1.34	0.98	1.85	**0.07**

RC = Reference Category

* P < 0.2

### 3.3. Result from mixed effect multivariable logistic regression

Table 4 presents the result of the mixed-effect multivariable logistic regression for demand and unmet need for contraception in four regions of Ethiopia. Preliminary analysis indicated that the ages of the women and their husbands had a Variable Inflation Factor (VIF) greater than 2.5. Therefore, these two predictors were excluded from mixed-effect multivariable logistic regression.

**Table 4:** Multivariable mixed effect logistic regression for demand and unmet need for contraception use, Ethiopia, N = 3343.

	Demand for Contraception use				Unmet need for contraception use			
	AOR	95%CI		P-value	AOR	95%CI		P-value
Random effect only model: EAs	**0.84**	**0.7**	**1.01**		**0.52**	**0.39**	**0.7**	
**Women work status**								
Not working ^RC^								
Working	1.34	1.10	1.62	0.00*				
**Women education status**								
Not able to read and write ^RC^								
Primary	1.92	1.53	2.42	0.00*	1.87	1.45	2.41	0.00*
Secondary and above	1.51	1.06	2.15	0.02*	1.21	0.80	1.83	0.37
**Household size**								
< = 5 members ^RC^								
>5 members					1.34	1.10	1.63	0.00*
**Husband’s education status**								
Not able to read and write ^RC^								
Primary	1.01	0.80	1.27	0.94	0.77	0.58	1.00	0.06
Secondary and above	1.06	0.79	1.40	0.71	1.14	0.83	1.55	0.42
**Household wealth index**								
Poorest ^RC^								
Poorer	1.44	1.07	1.93	0.01*	1.11	0.81	1.50	0.52
Middle	1.42	1.01	1.99	0.04*	0.69	0.47	1.02	0.07*
Richer	1.69	1.20	2.38	0.00*	0.69	0.47	1.01	0.06*
Richest	2.37	1.57	3.58	0.00*	0.51	0.32	0.81	0.00*
**Total children ever born**								
< = 3 ^RC^								
>3	0.83	0.69	0.99	0.05*				
**Husband working status**								
not working ^RC^								
working					0.68	0.54	0.87	0.00*
**Residence**								
Urban ^RC^								
Rural	1	0.63	1.58	0.99	0.94	-0.5	0.38	0.79
Number of groups = 220	AIC = 3734.36		BIC = 3813.71		AIC = 3020.02		BIC = 3099.37	
	ICC = 0.18		MOR = 2.23		ICC = 0.08		MOR = 1.65	
	Likelihood ratio = P value = 0.99				Likelihood ratio = P value = 0.80			

RC = Reference Category

Women’s working status, education status, household wealth index, and total number of children ever born were statistically significantly associated with the demand for contraception. Working women were 1.34 times more likely to demand contraception (AOR = 1.34; 95% CI (1.10,1.62)), P-Value = 0.00) compared to non-working women. Regarding education status; women who completed secondary education or higher were 1.51 times more likely to demand for contraception (AOR = 1.51; 95% CI (1.53, 2.42), P-Value = 0.02), and those who completed primary school were 1.92 times more likely to demand for contraception (AOR = 1.92; 95% CI (1.06, 2.15), p-value = 0.00) compared to the illiterate women. This study also revealed an increasing demand for contraception from the middle to richest wealth index categories: middle wealth index women were 1.42 times higher((AOR = 1.42; 95%CI (1.01, 1.99), P-Value = 0.04), richer wealth index women were 1.69 times more likely (AOR = 1.69; 95%CI (1.20,2.38), p-value = 0.00) and richest wealth index women were 2.37 times more likely (AOR = 2.37; 95% CI(1.57,3.58), p-value = 0.00 compared to women living in poorest wreath index households.

The ICC value in the final model for demand for contraception is 0.18, while the value of MOR and log-likelihood ratio result were 2.23 and 0.99 respectively, indicating that the model used in this study is the best fitting model.

The result in Table 4 also show that women’s education status, household size, wealth index and husbands’ employment status were statistically significantly associated with unmet need for contraception. Women with primary education were 1.87 times more likely to have an unmet need for contraception (AOR = 1.87; 95% CI (1.45, 2.41), P-value = 0.00) compared to illiterate women. Women living in households with more than five members were 1.34 times more likely to have an unmet need for contraception (AOR = 1.34; 95% CI (1.10, 1.63), P-Value = 0.00) compared to those in smaller households (five or fewer members). Additionally, women residing in richest wealth households had a lower likelihood (AOR = 0.51; 9%CI (0.32, 0.81), p-value = 0.00) of unmet need for contraception compared to those women living in the poorest wealth index category.

The ICC value was 0.08, and the results for MOR and Log-likelihood Ratio (LR) test were 1.65 and 0.80 respectively, for unmet need for contraception; these results also indicate the model was the best fit.

### 3.4. Result from concentration index

The results in Table 5 summarize the concentration index for the demand and unmet need for contraception in the four study regions in Ethiopia. The findings indicate that the demand for contraception was highly concentrated among women and their husbands with better educational status (at least having completed primary school and more), younger women, those living in households of five or fewer members, husbands with employment, and those residing in urban areas. Additionally, the results confirmed that the decrease in unmet need for contraception was significantly concentrated among working women, women in households of five or fewer members, and women with three or fewer children. Furthermore, the unmet need for contraception use was highly concentrated among women and their husbands who had attained an education level of primary school or higher.

**Table 5:** Concentration index for demand and unmet need for contraception use, four regions of Ethiopia (N = 3343).

	Demand for contraception use			Unmet need for contraception use		
	Index value	Robust std.	p-value	Index value	Robust std.	p-value
**Women age**						
15–24	0.3	0.05	**0.00**	-0.08	0.07	0.24
25–34	0.35	0.05	**0.00**	0.03	0.06	0.6
35–49	0.16	0.06	**0.01**	-0.03	0.07	0.64
**Women work status**						
Not working	0.26	0.05	**0.00**	0.01	0.04	0.77
Working	0.22	0.05	**0.00**	-0.15	0.06	**0.02**
**Women education status**						
Not able to read and write	0.21	0.04	**0.00**	0.02	0.05	0.67
Primary	0.15	0.06	**0.02**	-0.15	0.07	**0.02**
Secondary and above	0.15	0.07	**0.04**	-0.22	0.11	**0.05**
**Household size**						
< = 5 members	0.35	0.05	**0.00**	-0.08	0.05	**0.08**
>5 members	0.2	0.05	**0.00**	0.05	0.05	0.34
**Husband’s education**						
Not able to read and write	0.18	0.04	**0.00**	0.03	0.05	0.52
Primary	0.28	0.06	**0.00**	-0.12	0.06	**0.07**
Secondary and above	0.23	0.06	**0.00**	-0.14	0.07	**0.07**
**Total children ever born**						
< = 3	0.3	0.04	**0.00**	-0.14	0.05	**0.00**
>3	0.22	0.05	**0.00**	0.07	0.05	**0.17**
**Age of husband**						
15–24	0.3	0.11	**0.01**	-0.11	0.11	0.32
25–34	0.33	0.06	**0.00**	-0.06	0.06	0.34
35–45	0.26	0.05	**0.00**	-0.01	0.05	0.87
>45	0.19	0.08	**0.02**	0.02	0.09	0.81
**Husband work status**						
Not working	0.03	0.08	0.65	-0.01	0.08	0.91
Working	0.31	0.04	**0.00**	-0.02	0.04	0.65
**Residence**						
Urban	0.23	0.05	**0.00**	0.00	0.08	0.95
Rural	0.21	0.05	**0.00**	-0.01	0.04	0.83

### 3.5. Result from decomposition of concentration index

The results in Table 6 show the contribution of factors based on decomposition of the concentration index for the demand and unmet need for contraception use in four regions of Ethiopia. The findings indicate that the socioeconomic factors considered in this study played a significant role in the existing inequality in demand and unmet need for contraception among women in the study regions. Regarding the demand for contraception, illiteracy of the respondents (56%), wealth index/ richest economic status (41%), and non-working status of women (21%) contributed to a larger proportion of the variations in inequality. The highest elasticity was observed with respect to wealth index i.e., poorest (0.3060) and richest (0.1147), and women with three or fewer children ever born (0.1283). In terms of unmet need for contraception, the largest contributions to inequality is comes from the husbands’ illiteracy (197%) and household size of five or fewer members (184%). However, respondents’ illiteracy (-249%) and unemployment status (-119%) contributed to a decrease in inequality in unmet need for contraception. The maximum elasticity was detected in relation to the total number of children ever born, which is three or fewer (0.0892), and husbands’ illiteracy (0.0886).

**Table 6:** Contribution of factors based on decomposition of concentration index analysis of for demand and unmet need for contraception, four regions of Ethiopia, N = 3343.

	Demand for contraception				Unmet need for contraception			
	Elasticity	CI	Absolute contribution	percent contribution	Elasticity	CI	Absolute contribution	percent Contribution
**Women age**								
15–24	-0.0110	0.0331	-0.0004	0				
25–34	0.0102	-0.0196	-0.0002	0				
35-49^RC^								
**Women work status**								
Not working	-0.2273	-0.2620	0.0596	21	-0.0908	-0.2620	0.0238	-119
Working ^RC^								
**Women education status**								
Not able to read and write	-0.3193	-0.4952	0.1582	56	-0.1008	-0.4952	0.0499	-249
Primary	0.0000	0.3352	0.0000	0	0.0506	0.3352	0.0170	-85
Secondary and above ^RC^								
**Household size**								
< = 5 members	-0.1002	0.1567	-0.0157	-6	-0.2361	0.1567	-0.0370	184
>5 members ^RC^								
**Husband education**								
Not able to read and write	0.0649	-0.4454	-0.0289	-10	0.0886	-0.4454	-0.0395	197
Primary level	0.0328	0.1926	0.0063	2	-0.0206	0.1926	-0.0040	20
Secondary and above ^RC^								
**Total children ever born**								
< = 3	0.1283	0.2058	0.0264	9	0.0892	0.2058	0.0183	-91
>3 ^RC^								
**Age of husband**								
15–24	0.0059	0.0152	0.0001	0				
25–34	-0.0567	0.0338	-0.0019	-1				
35–45	-0.0587	-0.0597	0.0035	1				
>45 ^RC^								
**Husband working status**								
Not working ^RC^								
Working ^RC^	0.0718	0.1865	0.0134	5				
**Residence**								
Urban	-0.0287	0.8131	-0.0233	-8				
Rural ^RC^								
**Household wealth index**								
Poorest ^RC^								
Poorer	0.3060	0.2970	0.0091	3	0.0075	0.2970	0.0022	-11
Middle	0.0227	0.4939	0.0112	4	-0.0102	0.4939	-0.0051	25
Richer	0.0381	0.6606	0.0252	9	0.0040	0.6606	0.0027	-13
Richest	0.1147	1.0000	0.1147	41	-0.0335	1.0000	-0.0335	-3

## 4. Discussion

This study was primarily aimed at exploring inequalities in the demand for, and unmet need for, contraception among married/in-union and sexually active women in four regions (Afar, Benishangul-Gumuz, Gambella and Somali) of Ethiopia. The findings of the study revealed several prominent results regarding the individual, household, and community variables that predict the two outcome variables of the study.

The findings from the mixed-effect multivariable logistic regression for demand and unmet need for contraception indicate that women’s education had a positive association with both demand for and unmet need for contraception in the study regions. This finding is consistent with others studies conducted in different parts of the world, including research conducted in Saudi Arabia [35], Nigeria [36], Uganda [37], and various districts of Ethiopia [15, 16, 18, 38, 39]. Furthermore, the result of the decomposition of the concentration index signify that women’s education status is a significant contributor to the existing inequality in demand for and unmet need for contraception in the study regions. The most plausible explanation for this association could be that when women become educated, they gain better opportunities for autonomy and decision-making power regarding health service utilization. Additionally, educated women possess better knowledge about contraception choices, sources, benefit and possible adverse effects due to their access to printed media and information delivered in health facilities [15, 16, 38, 40, 41].

Interestingly, the study also revealed that illiterate women have lower unmet need for contraception than literate women, which is a finding consistent with research conducted in Nigeria [42] and Ethiopia [43]. While this result may seem counterintuitive at first glance, several [43] reasons could explain it. First, illiterate women may have limited access to information and services related to contraception, leading to lower awareness. Second, they may have less autonomy and decision-making power within their households, affecting their ability to use contraception even if desired. Third, differences in fertility preferences and norms between illiterate and literate women may contribute to varying levels of desire to avoid pregnancy.

Another noteworthy finding was the significant association between household wealth index and both demand for and unmet need for, contraception. Thus, better household wealth tends to increase the demand for contraception and decreases the unmet need for contraception. The decomposition of the concentration index further signifies that wealth index is one of the major contributors to the demand for family planning and a reduction of unmet need for contraception among women in the study regions. The finding is consistent with other studies conducted in Sub-Saharan African countries [44, 45] and a recent study in Ethiopia [39, 46]. If households have a higher wealth index, it may imply that they have access to different resources, giving them significant purchasing power [47, 48]. Given that the government may not have the capacity to provide all kinds of contraception methods in the required quantity and quality [46, 49], women in the poorest households may be limited in their access to family planning services as they typically cannot afford to visit private healthcare service providers/ pharmacies. In-addition, physical distances to health facilities may also impose additional transport cost on women residing in poor/poorest households [50].

Interestingly, the findings of this study revealed that working women were more likely to have a demand for contraception. Similarly, women with working husbands had a lower likelihood of having unmet need for contraception. Indeed, the decomposition of the concentration index also indicated the significant contribution of parental work status to the prevailing inequalities in demand for contraception among women. One plausible reason for this could be that raising or caring for children is one of the most demanding jobs, requiring time, money, and other resources. Given that most women in Ethiopia live in households with limited resources, some parents may decide to limit or space birth. Such decision is, in fact, an opportunity cost for the family to use the time and other available resources to improve existing family well-beings [51, 52]. Women’s employment status can also be seen as an increase in their freedom of movement and decision-making, which improves their ability to interact with friends and family [53].

The total number of children ever born is another significant predictor of demand for contraception, which also appeared as one of the largest contributors to inequality in the decomposition analysis. One possible explanation for the increased demand for contraception with higher parity is that, over time, parents tend to realize the economic and social burden that child care requires, such as increasing cost of education and a declined need for child labor [39, 54–56]. Furthermore, previous studies done by Melkalem et al. in Ethiopia indicate that women who have more than three children and are exposed to various insights regarding family planning utilization will have a very significant negative effect on unmet need for family planning [57].

A closely related variable, family size also had an important association with unmet need for contraception. Larger families typically have lower rates of unmet need for contraception. This is consistent with a previous study done in Nigeria which indicated that women who desired an extended family size were associated with unmet need for contraception compared to those who desired a smaller household size [42].

Decomposition analysis results indicated that paternal education had significantly larger contribution to inequalities in demand for, and unmet need for, contraception among women in the study regions. This is not surprising, as studies done in other regions of Ethiopia provide consistent conclusions. For instance, a study done in Butajira District, south-central Ethiopia [38], reported that husbands play a significant role in their wives’ use of contraception. One possible pathway for husbands’ higher education to influence family planning service utilization could be the likelihood of providing their wives with more freedom, support, and autonomy to make independent decisions [58–60]. Furthermore, more educated husbands provide ample opportunity to have good knowledge of limiting and spacing births, thereby promoting open discussion and communication with their wives. A study conducted in Ethiopia witnessed that educated husbands are more willing to engage their spouse in discussions about using contraception compared to illiterate husbands [38].

Finally, it is worth mentioning that the present study has strengths as well as some limitations. To start from its strengths; this study used representative data collected from all four regions of the study. The results of the study can be utilized by the authorities of those study regions as a basis for further planning, monitoring, and evaluation of family planning programs. In addition, this study employed a unique inequality analysis technique which provided more rigorous findings. Furthermore, the EDHS survey used internationally validated and standard data collection tools with slight modification to consider country specific contexts, along with well-designed standard procedures that ensured the validity and reliability of the collected data. The study is not immune to some limitations. One particular limitation we observed in this study is that EDHS used a cross-sectional design, which entails the collection of information at a specific period of time. This limits the ability of the study to make casual inferences. Also, some essential variables like husbands’ perception of contraception use and unmet need, and attitude towards different methods of contraception were not included in our analysis due to the limitations in obtaining adequate data.

## 5. Conclusions

This study has shown that there is a significant inequality in the demand and unmet need for contraception among women aged 15 to 49 years in the four study regions of Ethiopia. These regions are among the most disadvantaged in the country, according to the World Bank. However, our study also revealed that significant variations in demand and unmet need for contraception exist within these regions, depending on the socioeconomic characteristics of the women and their husbands. This suggests that even within the low-performing regions, there are groups of women who face more barriers and challenges in accessing and using family planning services than others. Therefore, we conclude that multiple layers of disparities affect the reproductive health outcomes of women in these regions, and addressing them requires tailored and targeted interventions that consider the specific needs and preferences of different subgroups of women. The findings imply an urgent need to narrow down the unacceptably high level of socioeconomic inequalities to reduce the unmet need for family planning and increase the demand for contraception in those parts of the country. The study strongly recommends the implementation of multidimensional and multisectoral approaches to significantly reduce inequalities in the two outcome variables.

## Supporting information

S1 Fig Concentration Index curve of women a) demand for contraception b) Unmet need contraception in four regions (Afar, Somali, Benishangul umuz, and Gambela) of Ethiopia. (DOCX)

S1 File Theoretical models selection and accounting for demographic differences in regression modelling. (PDF)
