# Supplementary material for: Inequalities in the demand and unmet need for contraception among women in four regions of Ethiopia
Source: PLoS One. 2024 Sep 10;19(9):e0308476. doi: 10.1371/journal.pone.0308476 (PMC11386449; doi:10.1371/journal.pone.0308476)
Supplement: S1 Fig — (DOCX) [file pone.0308476.s001.docx]

The concentration curve of this study also reveals that educated women have a higher demand for contraception, while uneducated women have a higher unmet need for it(See Fig 1).


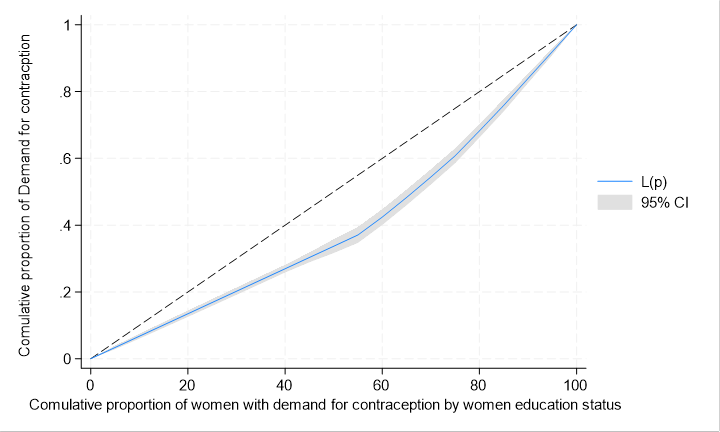


a) Demand for contraception


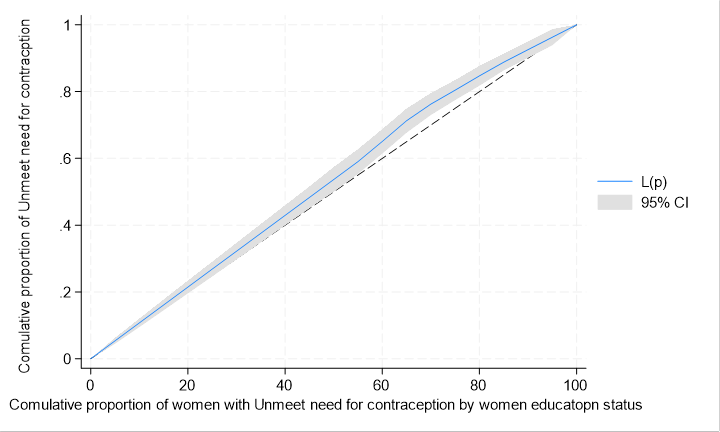


b) Unmet need contraception

**Fig 1:** Concentration Index curve of women a) demand for contraception b) Unmet need contraception in four regions (Afar, Somali, Benishangul umuz, and Gambela) of Ethiopia
