# Supplementary material for: Inequalities in the demand and unmet need for contraception among women in four regions of Ethiopia
Source: PLoS One. 2024 Sep 10;19(9):e0308476. doi: 10.1371/journal.pone.0308476 (PMC11386449; doi:10.1371/journal.pone.0308476)
Supplement: S1 File — (PDF) [file pone.0308476.s002.pdf]

Supplementary Material Two

Supplement #1: Theoretical Models Selection

This supplement provides additional context on how we arrived at the chosen theoretical models of the study.

1. **Literature Search Process:** To identify relevant theoretical models, we conducted an extensive literature search. We explored databases such as PubMed and Google Scholar using specific keywords related to health-seeking behaviour and social determinants of health. Our search terms included "health seeking behaviour," "social determinants of health," "theoretical model," and "framework." Additionally, we employed the snowballing technique, which involves examining references from retrieved articles to find additional relevant sources.
2. **Inclusion Criteria:** we screened the titles and abstracts of retrieved articles based on specific inclusion criteria:
    - The article should present or apply a theoretical model or framework related to health-seeking behaviour or social determinants of health.
    - The article must be published in English.
    - The article should be peer-reviewed. We also intentionally excluded studies that focused on specific diseases, interventions, or populations, aiming for models that are generalizable across different contexts.
3. **Evaluation and Selection:** After identifying potential models, we thoroughly assessed the full texts of relevant articles. Our evaluation involved comparing different models and frameworks based on their scope, components, and applicability to our research questions. As a result of this rigorous process, we opted for two well-established models:

    a) The Anderson Health Seeking Behavioural Model:
    - This model provides valuable insights into individual health-seeking behaviours.
    - It considers factors such as predisposing characteristics, enabling resources, and need factors.
    - By understanding these components, we gain a comprehensive view of how individuals seek healthcare.

b) **The Solar and Irwin's Commission on Social Determinants of Health Framework (CSDH):**
   - The CSDH framework takes a broader perspective, emphasizing social determinants that influence health outcomes.
   - It considers societal factors beyond individual behaviour, providing a holistic understanding of health disparities.

4. **Complementary Approach:** we believe that using both models enhances our understanding. The Anderson model focuses on individual behaviour, considering factors like predisposing characteristics, enabling resources, and need factors. In contrast, the CSDH framework takes a broader perspective, emphasizing societal factors that influence health outcomes. Together, these models provide a comprehensive and holistic view of the complex interactions between health-seeking behaviour, social determinants, and health status.

Supplement #2: Accounting for Demographic Differences in Regression Modelling

To address the demographic differences between respondents and background demographics across the four regions, we implemented the following strategies:

1. **Inclusion of Socio-Demographic Variables:**
   - We included relevant socio-demographic variables as independent predictors in our multivariable logistic regression model.
   - These variables encompassed age, employment status, education level, household wealth index, and place of residence.
   - Our selection was guided by the existing literature and the conceptual framework of our study.
2. **Mixed Effect Multivariable Logistic Regression:**
   - To account for clustering effects due to demographic variables, we employed a mixed effect model.
   - This approach estimates both fixed effects (independent variables) and random effects (demographic variables) on the outcome (unmet need for contraception).
   - The mixed effect model provides more accurate standard errors and confidence intervals compared to a standard logistic regression model.

3. **Addressing Multicollinearity:**
   - We conducted a preliminary analysis to detect multicollinearity among independent variables.
   - Notably, age of women and age of husbands exhibited significant multicollinearity (VIF > 2.5).
   - Consequently, we excluded these two predictors from the mixed effect multivariable logistic regression model.
